# Dr. Hedenus

**Published:** 1839-01

**Authors:** 


### DR. HEDENUS, OF DRESDEN.

Johann August Wilhelm Hedenus, the son of an apothecary, was born at Langensalza, in Thuringia, on the 11th August, 1760. He studied medicine at the Medico-Chirurgical College of Dresden, and, after passing through the necessary examinations, joined the army in 1783, as regimental surgeon. Here he remained two years, spending, however, one of these in the prosecution of his studies at Dresden. In 1791, he was appointed *Pensionairchirurg* and assistant prosector; and, two years afterwards, prosector of the Medico-Chirurgical College in that city. In 1798, he was promoted to *Generalstabchirurg*, and appointed professor of surgery, which office he held until 1808, when he was appointed body-surgeon to the king, and afterwards accompanied him on his travels. On his return, he came into great reputation and practice as a surgeon, and obtained in succession all those honorary distinctions which the medical men of Germany seem to prize so much. He was body-physician and body-surgeon to the king, government counsellor and medical counsellor, knight of the civil order of Merit, &c. &c. In 1824, Hedenus received the honorary degree of M.D. from the university of Leipzig. In July, 1833, the fiftieth anniversary of his public service, or, as it is called in Germany, his *Jubilee*, was celebrated at Dresden; on which occasion a medal was struck in his honour. He died at Dresden the 29th December, 1836.—Dr. Hedenus was a very accomplished surgeon, and, both by precept and example, greatly improved the state of surgical practice in Saxony. His mind was devoted chiefly to practical purposes, and accordingly he wrote fewer books than most of his distinguished brethren. As a surgeon, he was best known by foreigners on account of his operations for the extirpation of the thyroid gland, and for disease in the antrum of the upper jaw. The only distinct work we have from his pen is on this last subject: viz. "Antwort auf die Reclamation des Prof. Weinhold, meine Operations-und-Heilungsmethode eines Afterproducts der Highmorshöhle betreffend;" Leipzig, 1822.

